# A Silent Intruder: Unveiling a Schwannoma in the Distal Forearm

**DOI:** 10.7759/cureus.70771

**Published:** 2024-10-03

**Authors:** Mohd Hamdan Mohd Ibrahim, Shalimar Abdullah

**Affiliations:** 1 Orthopedics and Traumatology, Hospital Canselor Tuanku Muhriz UKM, Kuala Lumpur, MYS

**Keywords:** benign, forearm, median nerve, peripheral nervous tumour, schwannoma

## Abstract

Schwannomas are rare, benign tumors that develop from Schwann cells in the peripheral nervous system, and they make up only a small fraction of cases found in the upper limbs. Oftentimes, these tumors do not show symptoms until they start pressing on nearby structures. In this case report, we share the case of a 34-year-old woman who had painless swelling in her right distal forearm for three years. Unfortunately, this swelling eventually led to numbness and pain along the distribution of her median nerve. An MRI revealed a well-defined lesion associated with the median nerve, and a biopsy confirmed it as a schwannoma. After surgically removing the tumor, she experienced complete relief from her symptoms. This case underscores the importance of considering schwannomas when evaluating forearm masses and provides insight into the diagnostic and surgical approaches involved in managing these tumors.

## Introduction

Schwannomas, or neurilemmomas, are benign peripheral nerve sheath tumors that originate from Schwann cells [1]. While they constitute around 5% of soft tissue tumors in adults, these tumors are uncommon in the upper limb, accounting for just 19% of occurrences [2]. These tumors generally grow slowly and are often asymptomatic but may cause neurological symptoms as they expand, such as pain, numbness, or tingling due to nerve compression [3,4]. Schwannomas are typically well-encapsulated and tend to displace, rather than infiltrate the adjacent nerves [5,6]. A mass accompanied by numbness or pain, especially in the upper limb, warrants further investigation [5].

## Case presentation

A 34-year-old right-hand dominant lady, with no notable prior medical conditions, presented with a painless swelling in the volar aspect of the right forearm of three years duration with complaints of an increase in the size of the swelling associated with pain for the past three months. She complained of pain and paresthesia in the median nerve distribution of the right hand. Working as a staff nurse, these symptoms disrupted her regular activities and professional responsibilities. She has neither constitutional symptoms nor any family history of malignancy. She also denied any similar swelling elsewhere on her body.

During the physical examination, a 5 x 3 cm oval-shaped, firm, non-mobile mass was palpated on the volar aspect of the right distal forearm. A positive Tinel’s sign was noted upon percussion of the mass. Sensory evaluation indicated reduced responsiveness to light touch in the index finger, whereas motor function in the thumb remained unaffected (Medical Research Council scale 5).

**Figure 1:**
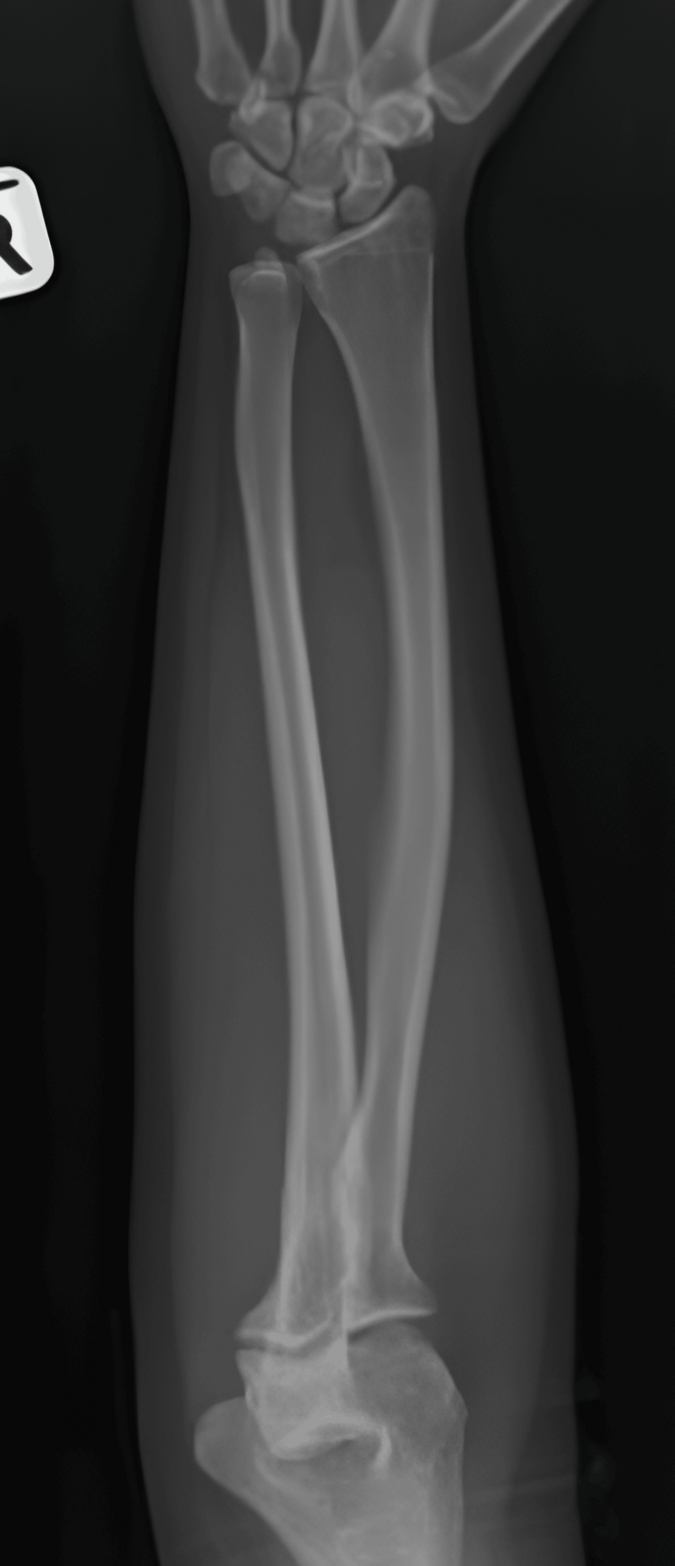
Plain radiograph of an anteroposterior view of the right forearm The radiograph of the right forearm shows no bony lesions or abnormal calcifications, with unremarkable surrounding soft tissues.

**Figure 2:**
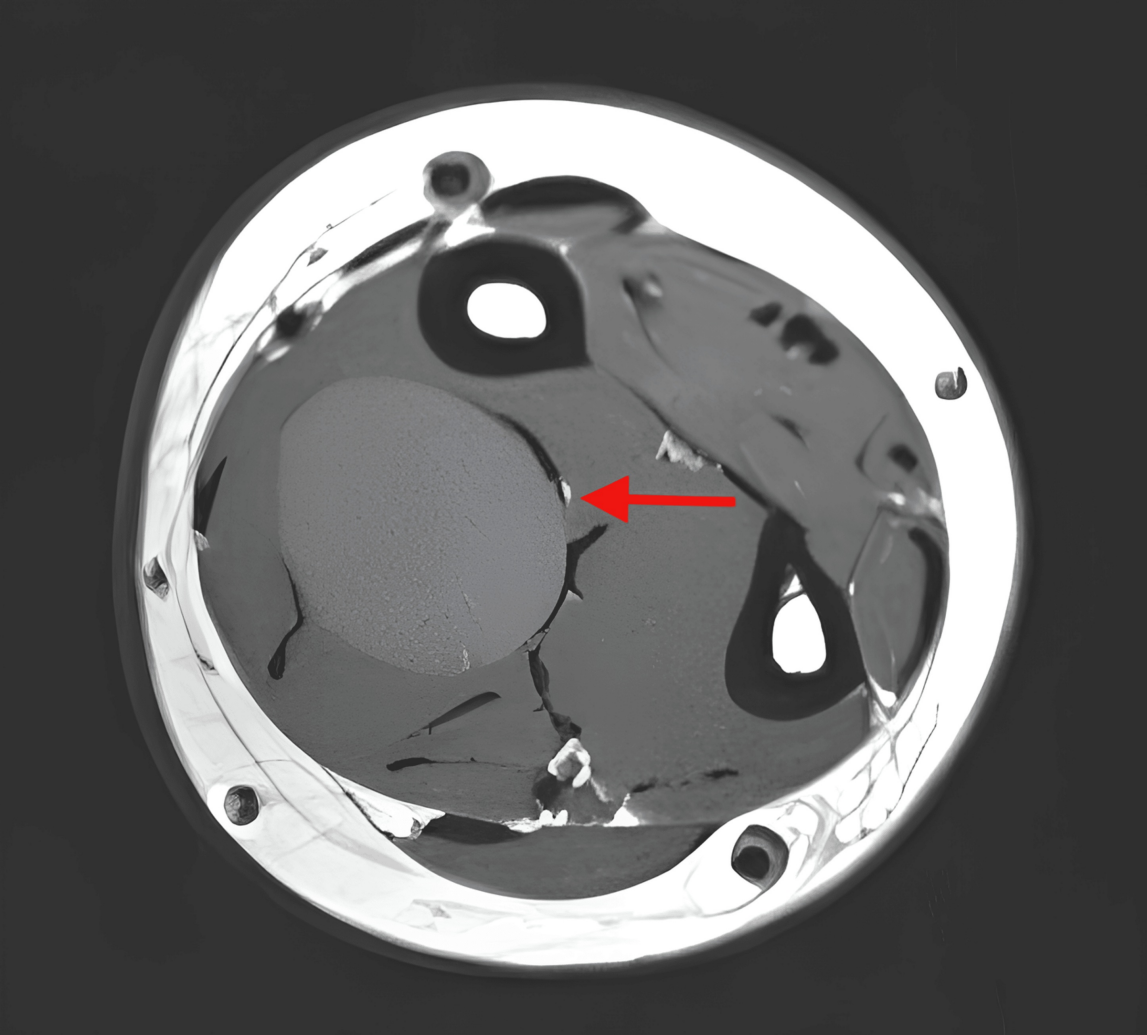
T2-weighted axial MRI image of the right forearm The red arrow shows a well-defined, oval-shaped heterogeneous hyperintense lesion in the volar compartment of the right forearm, with no infiltration into the adjacent tissues.

**Figure 3:**
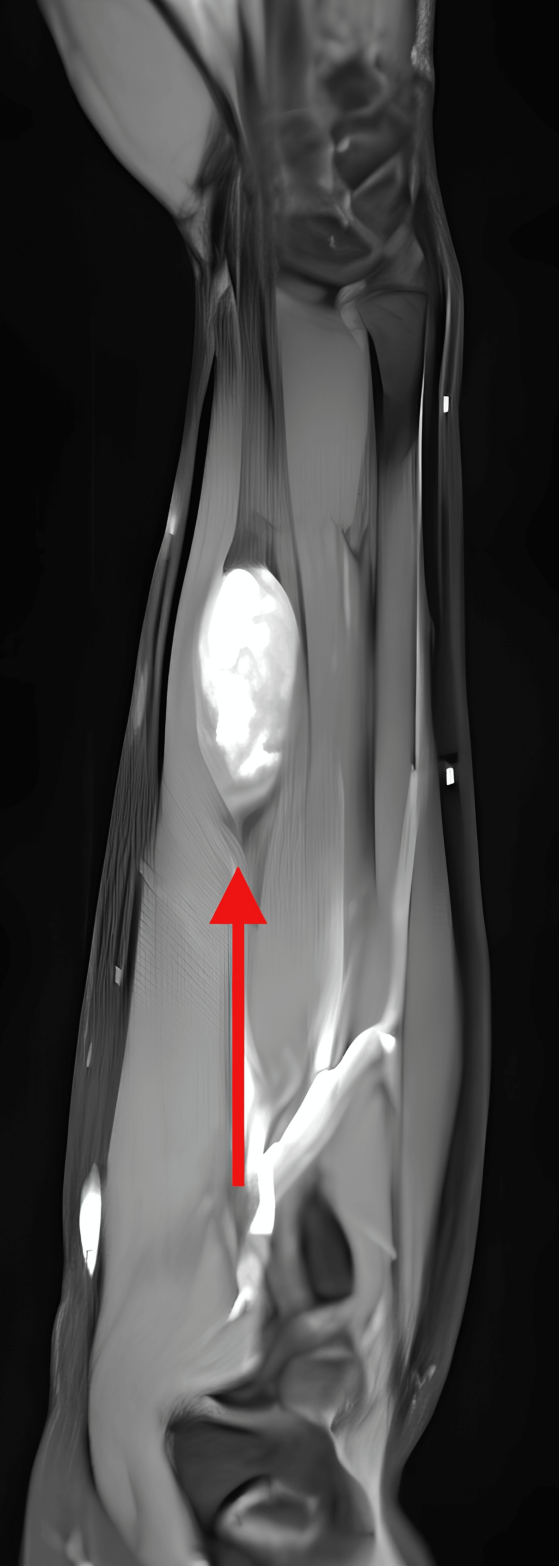
Fat-suppressed sagittal MRI image of the right forearm The red arrow shows a well-defined, oval-shaped, non-fat-suppressed, heterogeneous hyperintense lesion in the volar aspect of the distal right forearm, with no infiltration into the surrounding tissues.

The radiograph (Figure 1) revealed no osseous abnormalities or calcifications. The ultrasonography (USG) showed a well-defined heterogenous intramuscular lesion at the right anterior forearm, suggestive of the right forearm atypical intramuscular lipoma. To further evaluate the mass, an MRI of the right forearm was performed (Figures 2, 3). The MRI showed a well-defined heterogeneously hyperintense lesion on the T2-weighted imaging and a hyperintense lesion on the fat suppression sequences, measuring 1.8 x 1.9 x 4.4 cm, arising from the median nerve in the volar forearm. These findings were suggestive of the presence of a benign peripheral nerve sheath tumor, such as schwannoma or neurofibroma.

Based on the imaging results, an ultrasound-guided biopsy was conducted to confirm the diagnosis. Histopathological analysis revealed biphasic spindle cell characteristics, nuclear palisading, and hyalinized arteries, typical of schwannoma. Immunohistochemistry is frequently helpful in confirming the diagnosis, with the presence of S-100 protein serving as a crucial marker.

**Figure 4:**
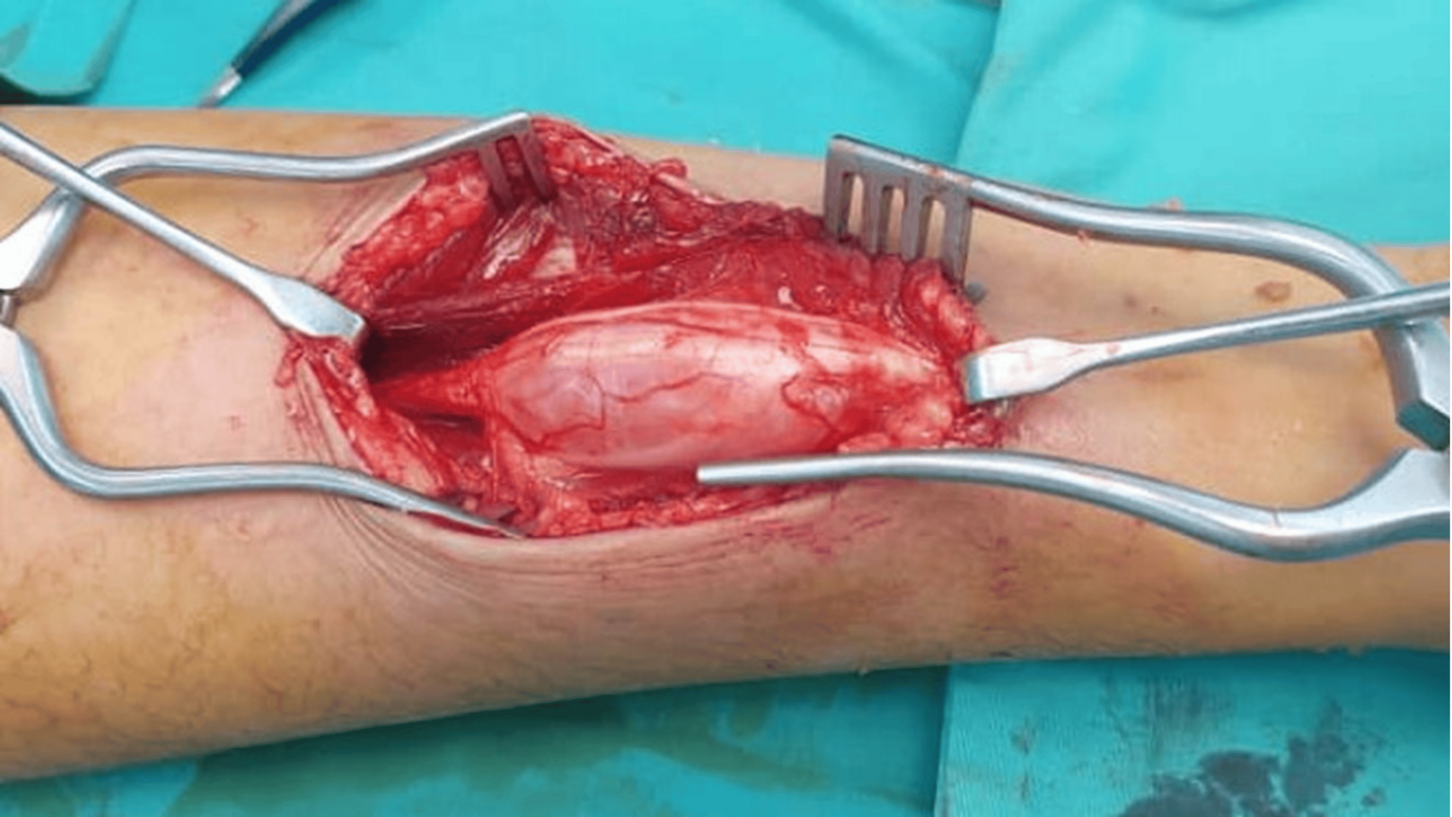
Intraoperative image of the volar aspect of the right forearm The intraoperative image shows the lesion splaying the median nerve.

**Figure 5:**
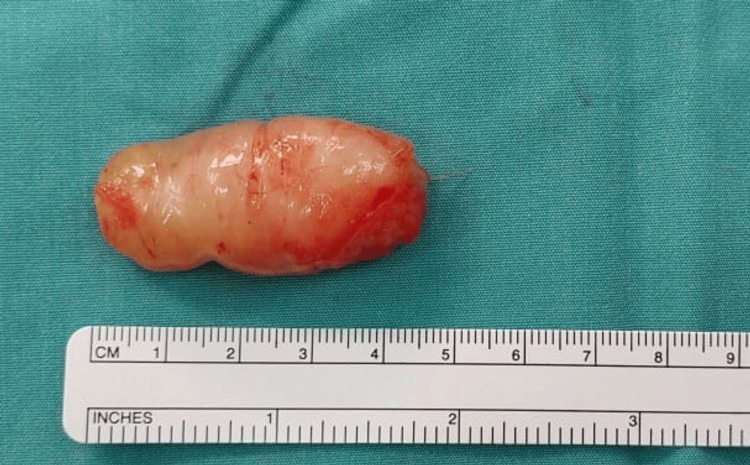
Lesion excised intraoperatively The intraoperative image shows a 4.0 x 1.5 cm firm yellowish lesion removed from the median nerve.

The patient was explained about the risks of the surgery, including the potential injury to the median nerve and the possibility of needing a sural nerve graft. A tumor-centered incision was made at the midline of the ventral surface of the left distal third forearm for adequate exposure of both the proximal and distal sides of the tumor on the median nerve. Upon exploration, it was discovered that the schwannoma had splayed the median nerve (Figures 4, 5). However, a complete excision of the tumor was successfully accomplished using microsurgical techniques without affecting the integrity of the median nerve. At her three-month follow-up, the patient reported complete resolution of numbness in the affected fingers and intact motor function.

## Discussion

Schwannomas may manifest as nodular, asymptomatic masses or cause neurological symptoms by compressing adjacent structures [1]. Although schwannomas are rare to occur in the forearm, this case highlights the need for physicians to consider this condition when evaluating forearm and wrist masses, particularly in cases when neurological symptoms are present [3,6]. Despite its infrequency, the involvement of the median nerve can result in sensory impairments, as shown in this particular case [7,8].

Magnetic resonance imaging (MRI) is the preferred imaging method for detecting schwannomas, as it is able to clearly define the size, location, and relation of the tumor to the surrounding tissues [3,6]. In this patient, the MRI revealed a clearly defined mass that appeared as isointense or slightly hyperintense on T1-weighted images and hyperintense on T2-weighted imaging. The aforementioned characteristics can aid in differentiating schwannomas from other types of soft tissue tumors [1].

Surgical excision is the standard treatment for schwannomas, particularly when the tumor results in discomfort, numbness, or functional impairment [2,3,6]. Schwannomas possess a high degree of encapsulation, enabling their full removal while maintaining nerve activity [1]. Conversely, neurofibromas, which are a different form of peripheral nerve sheath tumor, display a higher degree of infiltration and may necessitate more intricate surgical treatment [1,6]. In this case, surgical excision resulted in the total elimination of symptoms without any motor or sensory impairments after the operation. To prevent nerve fiber damage during both epineural and endoneurial dissection, surgeons must perform meticulous microsurgical dissection in a bloodless surgical field with the use of magnification. Surgeons must exercise caution to avoid unnecessarily sacrificing functionally essential motor and sensory branches, as paraesthesia is the most frequently reported complication following surgery [9]. Long-term follow-up is crucial to monitor for recurrence, although the prognosis following excision of benign schwannomas is generally excellent [4,8].

## Conclusions

Peripheral nerve schwannomas can be difficult to diagnose as they can present with symptoms similar to those of lipomas, ganglions, fibromas, or xanthomas. This case study demonstrates the successful treatment of a rare schwannoma in the distal forearm. It also shows how important it is to include schwannomas in the differential diagnosis of forearm tumors, especially when neurological symptoms are present. For a correct diagnosis, a thorough clinical and radiological assessment is necessary, with appropriate imaging and biopsy being key components. The best course of action is still surgical excision, which produces good results when done with proper microsurgical methods that protect the parent nerve. The observation of individual cases like this contributes to a better understanding of the etiology, pathogenesis, and management of this rare disease.
